# Prevalence of Lumbar Segmental Instability in Young Individuals with the Different Types of Lumbar Disc Herniation—Preliminary Report

**DOI:** 10.3390/ijerph19159378

**Published:** 2022-07-31

**Authors:** Tomasz Kuligowski

**Affiliations:** Faculty of Physiotherapy, University School of Physical Education in Wroclaw, 51-612 Wrocław, Poland; tomasz.kuligowski@awf.wroc.pl

**Keywords:** spine, segmental instability, lumbar disc herniation, low back pain

## Abstract

Lumbar segmental instability (LSI) can cause pain and disability, and its background can be related to lumbar disc herniation (LDH). This retrospective study was conducted to analyze the prevalence of lumbar segmental instability (LSI) in young patients with different types of lumbar disc herniation (LDH). The study evaluated 133 individuals (18–25 years old) who suffered from LDH and underwent MRI and flexion-extension X-rays. Two groups were created: protrusion (PRO) and extrusion (EXT). LSI was scored positive when translatory motion was greater than 4 mm anteriorly or 2 mm posteriorly at the level of herniation. Statistica 13 was used to perform statistics. The LSI overall prevalence was 18.33% in PRO and 21.92% in EXT (*p* > 0.05). Out of all LSI positives, higher LSI incidence was observed in females compared to males; in PRO: 63.64%; in EXT: 68.75% (*p* > 0.05). LSI correlated positively with the passive lumbar extension test (PLE) (R = 0.32; *p* = 0.01) in the PRO group only. In summary, the results showed that the overall incidence of LSI was higher with severer disc damage. In addition, females were more prone to this pathology. However, the different types of LDH do not significantly affect the prevalence of LSI in young individuals.

## 1. Introduction

Low back pain (LBP) is one of the most common causes of adult disability. The mean age of people experiencing this pathology is consecutively lowering. LBP can result from many disorders, including intervertebral discs, ligaments, facet joints, nerve roots, and paraspinal muscle pathologies. A lumbar segmental instability (LSI) might be one of the causes of LBP. According to the literature, its prevalence can be as high as 57% of patients with chronic low back pain [1]. The differentiation diagnostics play a crucial role in clinical reasoning, and choosing the most appropriate treatment method that could be beneficial for specific patients is required [2]. Research on LSI has primarily focused on determining its definition, background, symptoms, and prevalence among non-specific LBP patients [3,4,5]. The LSI is related to the proper and balanced working of the three subsystems: active, passive, and neural control [6]. Abnormal function of one of them can lead to an overload of others and cause pain and reduced quality of life [7].

Nevertheless, the LSI definition remains challenging to unify [8]. Typical LSI symptoms are local pain during standing for an extended period, painful passive and active lumbar flexion/extension, tenderness of subcutaneous tissue (especially ligamentous), tendency to lean forward within the center of pressure (CoP) of the body in relation to poor core control, and painful position changing [9]. Those symptoms might lead to further injury or spondylolisthesis [10]. Other authors also describe the functional type of instability of the lumbar spine, which can proceed without any structural abnormalities observable in radiological examination [10,11]. This mechanism could possibly be also related to young individuals, but it is challenging to find any data in the literature. It should be mentioned that the total lumbar ROM might also be affected by the upper parts of the spine, such as thoracic scoliosis. Further compensations or treatment results could lead to low back pain [12,13]. Another division was proposed by Areeudomwong et al. for clinical and radiological lumbar instability (CLI and RLI) [14]. However, those two mechanisms overlap.

One of the hypotheses of LSI background is a pathology of the lumbar intervertebral disc (LIVD). LIVD can damage throughout life by gradual dehydration or react to an excessive load with damage: protrusion or extrusion. Consequently, herniated disc has reduced height which also reduces the interbody distance. However, longitudinal ligaments’ length remains unchanged. This approach has been previously suggested in the literature [15,16,17]. Such a mechanism can lead to increased three-dimensional segmental motion causing symptoms of the LSI. Thus, little to no information can be found in the literature on the prevalence of LSI in different types of LDH, especially in young people; therefore, the total range of motion (ROM) in the spine and other joints decreases throughout life. On the other hand, protrusion and extrusion of the disc have clinical and radiological differences, some of which might be related to functional outcomes [18].

Most authors agree on the diagnosis of LSI. It is usually confirmed using a flexion-extension radiograph [19] to determine the translation. However, different radiological criteria have previously been proposed. As the spondylolisthesis stages seem to be commonly accepted (1st stage with at least 25% of vertebral body translation), in this study, we followed the values proposed by Panjabi and White—4 mm of anterior translation or 2 mm posteriorly in the sagittal plane [9]. To complete and confirm the diagnosis, several clinical tests were found to help determine the LSI, such as the passive lumbar extension test (PLE), the rocking test (RT), lumbar quadrant tests (QL), painful catch sign and some the manual examination maneuvers (passive physiological intervertebral movement—PPIVM) [2,14,16,19,20]. Treatment of this pathology is generally conservative and focuses on improving core neuromuscular control, focusing primarily on the transverse abdominal muscles (TrA) and the lumbar multifidus muscles (LM) [21,22,23]. Surgical options could be considered only in carefully selected patients [24].

To date, lumbar instability has been subject to constant research. Not all mechanisms of the LSI have yet been fully understood. To our knowledge, no one has so far studied determining the prevalence and distribution of the LSI in different types of LDH in young individuals, particularly those under the age of 30. Most authors accepted a wide age range which could have given disturbing observations. Thus, this study aims to clarify the prevalence of LSI among young people with a different type of LDH.

## 2. Materials and Methods

### 2.1. Participant Selection

**Figure 1:**
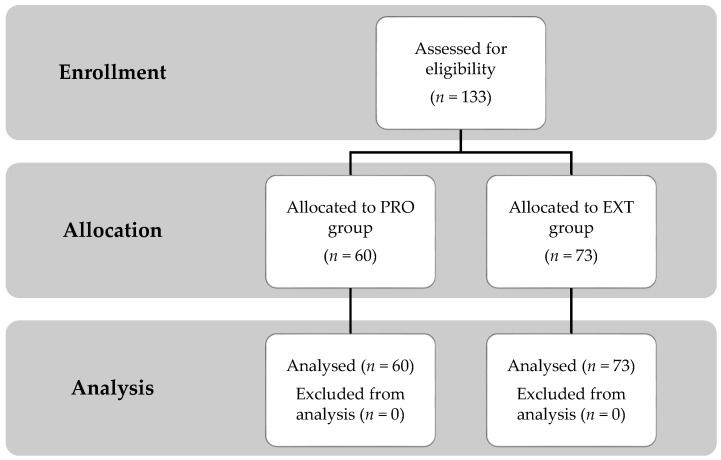
Participant allocation chart.

In this case, 133 adults between 18 and 25 years who were patients of a private physiotherapy clinic in Wroclaw, Poland, between January 2018 and December 2021 were randomly selected for this retrospective cohort study. Before enrollment, every participant underwent magnetic resonance imaging (MRI) and flexion-extension X-ray. Additionally, clinical examination and functional outcomes were performed, including the Oswestry Disability Index questionnaire (ODI), Numeric Pain Rating Scale (NPRS), Passive Lumbar Extension test (PLE), and Painful Catch Sign. The lumbar herniated disc diagnosed by MRI was the primary condition. All criteria for selecting patients were as follows: age between 18 and 25 years, the presence of single-segment lower lumbar herniated disc (protruded or extruded), local pain for at least 12 weeks during either lumbar flexion or extension, subjective pain level of at least 4/10 using NPRS. Subjects were excluded if they confirmed the presence of any of the following: uni- or bilateral leg symptoms, pelvis or spine area surgical history, polyneuropathy, spinal stenosis, prior spinal fracture, structural scoliosis, cauda equina syndrome, spondylolisthesis, or contraindications to radiological assessment. All subjects who met inclusion criteria were divided into one of the two groups depending on the type of the LDH (according to North American Spine Society—NASS): protrusion (PRO, *n* = 60) or extrusion (EXT, *n* = 73) (Figure 1).

The same physiotherapist with more than 10 years of experience (certified manual therapist) performed all the examination procedures. All MRIs and X-Rays were carried out by the same external, specialized medical center. A radiologist interpreted every scan.

The study was approved by the institutional ethics committee of the University School of Physical Education in Wroclaw, Poland. Written informed consent was obtained from all participants to use their medical data and images.

### 2.2. Radiographic Outcome Measure

Flexion-extension X-rays were taken according to routine principles. The lateral projection was obtained in maximum flexion and extension of the lumbar spine from a standing position with arms crossed on the shoulders and extended knees with feet set to the width of the pelvis during the whole process. MRI scans were performed by guidelines. All measurements were completed by a radiologist blinded to the other clinical test results. The LSI radiological criteria were set as suggested by Panjabi and White to be at least 4 mm of translatory motion in the sagittal plane anteriorly or 2 mm posteriorly [9]. Cases that have met one of these criteria at the same level as the LDH have been scored positive.

### 2.3. Lumbar Disc Herniation

**Figure 2:**
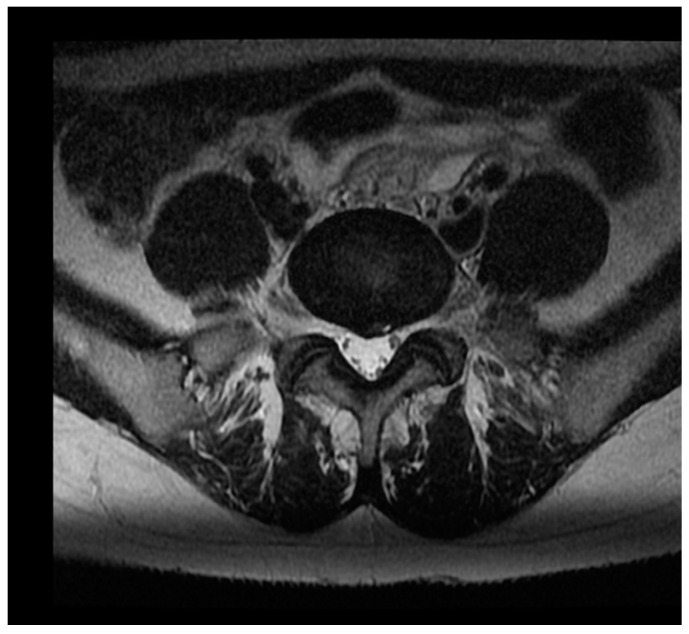
An example image of intervertebral disc protrusion in MRI (1.5 T, T2 transverse planes).

**Figure 3:**
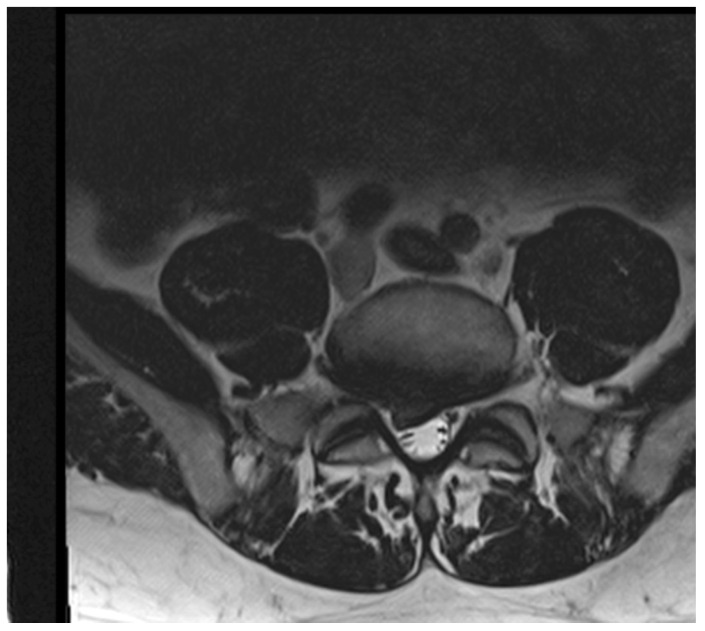
An example image of intervertebral disc extrusion MRI (1.5 T, T2 transverse planes).

A herniated disc is described in detail by the North American Spine Society [25]. The proposed division for protrusion (Figure 2) and extrusion (Figure 3) depending on radiological measurement was used. A protrusion is when a disc material displacement of more than 25% of the disc space, with the largest measure, in any plane. On the other hand, an extruded disc is described as when the largest measurement of the displacement disc material is greater than the base of the disc material at the disc space of origin if measured at the same plane. Another commonly described disc characteristic is “bulging”, which according to the literature, is not considered herniation. All measurements were completed by two radiologists who were blinded to the other clinical test results and each other.

### 2.4. Oswestry Disability Index

The ODI questionnaire was used to subjectively assess the level of patients’ disability [26]. This is one of the most commonly recommended condition outcome tools for spinal dysfunctions. It contains 10 sections relating to daily activities, each scoring 0–5 points, where 0 represents no disability and five the most significant disability. The total score is interpreted as follows: minimal disability (0–20%), moderate disability (21–40%), severe disability (41–60%), crippled (61–80%), to the bed-bound (81–100%). This questionnaire was used in the Polish language version.

### 2.5. Passive Lumbar Extension Test

This test was performed according to the suggestions of its authors [27]. The subject was in a prone position, and then the two lower extremities with knees actively extended were elevated at about 30 cm from the bed simultaneously. It was scored as positive if it provoked the patient’s symptoms and was immediately stopped when it did so. The process was repeated three times to avoid a false-positive result.

### 2.6. Painful Catch Sign

The test was carried out following the literature’s suggestions [14,20,28]. The patient was positioned supine with the lower extremities extended in the knee joints, then was asked to lift the legs and slowly return to starting position. Any movement disturbances due to pain in the lower back were judged as LSI. If it caused symptoms for the patient, it was considered positive and stopped when it happened. The test was repeated three times to avoid false-positive results.

### 2.7. Data Analysis

All statistical analyses were performed using Statistica 13 software (StatSoft, Kraków, Poland). Data were presented as the mean and standard deviation (SD) or median and interquartile range (IQR). Descriptive statistics were performed to display the demographic data and prevalence of LSI in the population of young individuals with LDH. Differences in demographics between participants between the PRO and EXT groups were examined by applying the Mann–Whitney U test or independent T-test, depending on the data distribution. For dichotomous values (PLE, Painful catch, and others), the Chi^2^ test with optional Yates correction was used. Correlations were calculated using Spearman’s test. Significance levels were set at *p* ≤ 0.05.

## 3. Results

In total, 133 participants were screened in this study (71 females and 62 males, 60 with protruded and 73 with extruded lumbar disc, 22.68 ± 2.10 years, 172 ± 8 cm, 67.29 ± 10.91 kg). The sample size at each stage is presented in Figure 1. Participants’ demographics are shown in Table 1. There were no significant differences between the groups.

An additional correlation was performed and presented in Table 2. A stronger relation coefficient was observed within most of the variables in the EXT group compared to PRO; however, the most reliable statistical aspect value was Painful Catch vs. PLE (0.47 vs. 0.67, respectively, both *p* = 0.00). LSI correlation was significant vs. PLE only in the PRO group (0.01), while in the EXT group, values were not statistically significant (0.07). In the EXT group, no correlation was observed between LSI and PLE either Painful Catch. In the studied sample size, stronger relation was observed between instability tests than between the presence of the LSI and clinical tests. Correlation data was presented in Table 2.

The overall prevalence of the LSI was higher in the EXT group (PRO 18.33% and EXT 21.92%); however, these values did not represent the population, neither divided by sex nor without (*p* = 0.76, 0.45, 0.60, respectively). In addition, the sample size showed an increased incidence of the LSI in females (in PRO: 63.64% of LSI presence were females; in EXT: 68.75% of LSI presence were males); thus, gender did not influence the prevalence of the LSI between the groups (Table 3).

Another analysis was made to determine the possible LDH influences on the LSI, including only ‘positive’ LSI participants. Although the pain level, subjective disability questionnaire values, and positive painful catch test frequency were slightly higher in the extrusion group, the overall results (Table 4) show that the LDH type did not affect the LSI significantly in terms of any of the analyzed parameters.

## 4. Discussion

This study appears to be the first to investigate the influence of the different types of lumbar herniated disc (protrusion or extrusion) on the prevalence of LSI that covered only young adults (age range 18–25 years) with single-segment disc damage combined with clinical and functional outcome. While an overall prevalence of LSI can be identified in up to 57% of people with non-specific low back pain (nLBP), there is little to no information in the literature about the prevalence of LSI in young people with LDH. Thus, the study aimed to determine whether the different types of lumbar disc herniation could influence the prevalence of LSI in young adults.

Although the literature suggests that the translational segmental motion can increase gradually with the damage [29], in most cases, this paper’s results are generally consistent with most previous works, and no significant relationship was found between the LSI and the LDH types. Some trends are seen between the groups (higher percentage of the LSI with extruded disc), but the differences do not mirror the population statistically. An explanation for no significance might be challenging. Other authors observed that despite the motion of adjacent segments to the segment with LDH mainly remaining unchanged, the segmental motion of the LDH disc is usually disturbed [30]. Moreover, Keorochana et al. suggested that the segmental motion also depends on the spinal alignment and the specific level of herniation [31]. The current study confirmed the higher percentage of LSI incidence among the extrusion group compared to the protrusion. This mechanism was previously described and is probably related to decreased interbody distance with relatively more ligament laxity within the specific segment, as was also proposed by other authors [15,16,17]. Consequently, Kong et al. also confirmed that an increased translational motion is related to lumbar disc damage. They also suggested that an important role can be played by ligamentum flavum, which hypotrophy might be a reason for more translatory segmental motion [32].

A similar study was conducted by Bram et al. They researched determining the influence of MR spinal abnormalities on segmental instability, including intervertebral disc (IVD) pathologies. No significant results were observed between LDH and LSI; however, 60 patients widely age ranged (19–83 years old) were screened, where other abnormalities related to bone-marrow, endplate and multi-level IVD were accepted as inclusion criteria [33]. Instead, the current study’s results showed an increased frequency of LSI presence with extruded lumbar disc compared to protrusion, which was also observed by Kong et al. [34]. Murata et al. observed the increased incidence of LSI concerning lumbar disc degeneration in up to 32.1% of participants. However, no significant correlation was found between a tilting movement and a horizontal displacement (translatory motion)—although 17 out of 107 L4-5 discs were displaced at least 3 mm [35]. Their group consisted of 109 individuals, similarly to Bram et al., widely age ranged (from 14 to 82 years old), which might have disturbed the results somehow. Still, the above authors focused mainly on degenerative disc disease (DDD) rather than LDH (consistently described by NASS and ASNR) in young adults, as was proposed in this research.

This study showed a relatively weak correlation between the clinical instability tests (PLE and Painful Catch) and the presence of LSI in flexion-extension radiographs. Painful Catch sign had stronger relation in the extrusion group, but statistically, it was only borderline of significance (*p* = 0.05). PLE test’s correlation coefficient was positive; it was also significant in the protrusion group (*p* = 0.05); however, its value (r = 0.32) should be interpreted with care because it might be too little in the medical sciences according to statistical manner. Hence, it may be challenging to confirm the LSI in LDH patients based only on clinical tests, which is crucial in choosing the most appropriate treatment for a specific patient. These results, however, need to be interpreted with caution. That is because there is little to no information about the differences between LSI symptoms in LDH versus nLBP patients, and this paper performed no likelihood ratios (LRs) nor other suggested calculations as this was not the aim of the current study. Especially regarding the clinical outcome and the efficiency of the LSI tests between those pathologies. Nonetheless, other authors proved the clinical efficacy of those tests in their studies [14,20].

Similar to Fujiwara et al., the present study shows no significant gender differences regarding the occurrence of the LSI [36]. Although the percentage incidence was higher among women, the results were not statistically significant. It is very plausible that a larger sample size would have given more variable results in future research because more ligamentous laxity is often observed in females.

### Study Limitations

It should be highlighted that there were some limitations to the study, which should be considered in future prospective research. First, although the inclusion criteria were quite restricted, the prevalence of the LSI was smaller than expected. Its incidence probably was affected by pain occurring during flexion-extension radiographs, which could not allow the participant to obtain a full range of motion. Thus, the position was determined by the X-Ray device and radiologist. Moreover, the participants’ sports activity level was not assessed, which could affect the total lumbar spine ROM. Secondly, the sample size may need an improvement—a higher percentage of LSI individuals should be examined in further research. Thirdly, sagittal spinal alignment was not evaluated, which might have been a significant matter within the LDH pathology and biomechanics.

## 5. Conclusions

In conclusion, this study showed that the different types of LDH do not affect the prevalence of LSI in young individuals. In addition, it showed that gender type does not affect the LSI prevalence, nor the clinical outcome of the LSI patients was found to be related to the LDH. While the tendency for a higher percentage of the LSI incidence and higher values of ODI, NPRS and Painful Catch Sign in the extrusion group were observed, no statistical significance was achieved. As the LSI might be present in 1/5 of young people with LDH, the PLE and Painful Catch sign tests or flexion-extension radiographs can be worthy of consideration in the early stage of clinical reasoning and treatment planning.

## Figures and Tables

**Table 1:** Subjects’ demographic and clinical characteristics.

Variable	PRO (*n* = 60)	EXT (*n* = 73)	*p*
Age (years)	22.47 (2.30) [4.00]	22.85 (1.92) [2.00]	0.49
Sex (female/male)	33/27 [1.00]	38/35 [1.00]	0.77
Body mass (kg)	67.32 (10.23) [0.13]	67.27 (11.51) [0.09]	0.79
Body height (cm)	172 (0.08) [17.00]	171 (0.08) [18.00]	0.15
BMI (kg/cm^2^)	22.52 (2.00) [2.68]	22.94 (2.64) [4.15]	0.30
ODI (0–100)	30.03 (7.32) [11.00]	30.85 (9.19) [16.00]	0.58
NPRS (0–10)	4.85 (1.01) [1.00]	5.35 (2.67) [2.00]	0.86
PLE (%, *n*)	48.33 (29) [1.00]	54.79 (40) [1.00]	0.52
Painful Catch sign (%, *n*)	48.33 (29) [1.00]	60.27 (44) [1.00]	0.24
LSI (%, *n*)	18.33 (11) [0.00]	21.92 (16) [0.00]	0.72

BMI—body mass index; ODI—Oswestry Disability Index; NPRS—Numeric Pain Rating Scale; PLE—passive lumbar extension test; SLR—straight leg raise test; PRO—protrusion group; EXT—extrusion group; LSI—lumbar segmental instability; values are expressed as ‘*n*’ or Mean (SD) or median and [IQR].

**Table 2:** Correlations between clinical instability parameters.

Variables	Group	R Spearman	*p*
Painful Catch vs. PLE	PRO (*n* = 60)	0.47	0.00
Painful catch vs. LSI		0.15	0.27
LSI vs. PLE		0.32	0.01
Painful Catch vs. PLE	EXT (*n* = 73)	0.67	0.00
Painful catch vs. LSI		0.23	0.05
LSI vs. PLE		0.22	0.07

PLE—passive lumbar extension test; LSI—lumbar segmental instability; PRO—protrusion group; EXT—extrusion group.

**Table 3:** The prevalence of the LSI in individuals with the different LDH type.

	PRO (*n* = 60)		EXT (*n* = 73)		χ^2^, *p*
	With LSI	Without LSI	With LSI	Without LSI	
Male	4 (14.81%)	23 (85.19%)	5 (14.29%)	30 (85.71%)	0.09 *, 0.76 *
Female	7 (21.21%)	26 (78.79%)	11 (28.95%)	27 (71.05%)	0.55, 0.45
Total	11 (18.33%)	49 (81.67%)	16 (21.92%)	57 (78.08%)	0.26, 0.60

PRO—protrusion group; EXT—extrusion group; LSI- lumbar segmental instability; LDH—lumbar disc herniation; χ^2^—chi-square test; * Yates correction.

**Table 4:** Differences between the groups within the LSI ‘positive’ participants only.

Variable	PRO (*n* = 11)	EXT (*n* = 16)	*p*
ODI (0–100)	31.45 (6.58) [12.00]	34.63 (8.75) [11.00]	0.23 *
NPRS (0–10)	5.82 (0.87) [2.00]	6.44 (1.46) [3.00]	0.27 *
PLE (%, *n*)	81.82 (9) [-]	75.00 (12) [-]	0.79 *
Painful Catch sign (%, *n*)	63.64 (7) [-]	81.25 (13) [-]	0.45 *

ODI—Oswestry Disability Index; NPRS—Numeric Pain Rating Scale; PLE—passive lumbar extension test; PRO—protrusion group; EXT—extrusion group; LSI—lumbar segmental instability; * Mann-Whitney U test; values are expressed as ‘*n*’ or Mean (SD) or median and [IQR].

## Data Availability

Not applicable.
